# An evaluation of solid versus liquid transport media for high-risk HPV detection and cervical cancer screening on self-collected specimens

**DOI:** 10.1186/s13027-020-00333-4

**Published:** 2020-11-30

**Authors:** Hui Du, Xianzhi Duan, Yan Liu, Bin Shi, Wei Zhang, Chun Wang, Xinfen Qu, Juncui Bao, Jingran Li, Chao Zhao, Jing Jiang, Juan Liu, Kejia Wu, Aimin Xiao, Lvfang Duan, Xia Huang, Shuhuang Bian, Lijie Zhang, Hongxue Luo, Lihui Wei, Jerome L. Belinson, Ruifang Wu, CHIMUST group

**Affiliations:** 1 Peking University Shenzhen Hospital, Shenzhen, PR China; 2 Capital Medical University Beijing Tongren Hospital, Beijing, PR China; 3 Fudan University Huashan Hospital North, Shanghai, PR China; 4 The second Hospital of Hebei Medical University, Shijiazhuang, PR China; 5 Wuhan University Zhongnan Hospital, Wuhan, PR China; 6 Peking University People’s Hospital, Beijing, PR China; 7 Preventive Oncology International Inc, and the Cleveland Clinic, Cleveland, OH USA

**Keywords:** Cervical Cancer screening, High risk HPV testing, Solid media transport, Self-collection

## Abstract

**Background:**

The solid transport media is a small size card that allows fast, easy DNA extraction from a variety of biological samples. In 2016 we developed a solid media transport card; for that pilot study to control the self-collection we used a pseudo-self-collection technique. The current study expands this prior work using true self-collections and only the POI card, and aims to evaluate the solid media transport card to detect HR-HPV in self-samples compared to liquid transport media.

**Methods:**

Ten thousand eight hundred eighty-five women between the ages of 30–59 with no screening for 3 years were enrolled. The self-collected sample was first applied to a new solid media transport card (Labeled as SC) then the brush placed in 6 ml ThinPrep liquid (Labeled as SL). Then a physician collected a direct endocervical specimen into ThinPrep liquid (Labeled as DL). Samples were tested with Cobas 4800 and the SeqHPV NGS assay for HR-HPV. Patients positive on any test were recalled for colposcopy and biopsy.

**Results:**

Ten thousand three hundred thirty-nine participants had complete data. The mean age was 43.9 years. CIN 2+ rates were 1.4% (142/10339). The agreement in HPV detection between the two different self-sample collection media was also good (Cobas HPV kappa = 0.86; SeqHPV kappa = 0.98). Tested with Cobas, the sensitivity of Cobas-SL and Cobas-SC for CIN 2+ was95.07 and 94.37%; and for CIN3+ was 96.30, 96.30% respectively. The specificity of Cobas-SC, and Cobas-SL for CIN2+ was 88.74 and 87.35%; for CIN3 was 88.04and 86.65% respectively. Tested with SeqHPV, the sensitivity for CIN2+ of Seq-SC and Seq-SL was 95.77 and 96.48%; for CIN3+, both the SC and SL specimens had a sensitivity of 100%. The specificity for CIN2+ of Seq-SC and Seq-SL was 89.54 and 89.53%; for CIN3+ was 88.84,88.82% respectively. For both HR-HPV assays, the sensitivities were similar for the two self-sample media (SC vs SL, *p* = 1.00).

**Conclusions:**

The solid transport card for collecting vaginal self-samples as accurate as liquid transport media assayed by two different PCR based HR-HPV tests. The solid transport media is a suitable medium for collecting and storing vaginal self-samples.

**Supplementary Information:**

The online version contains supplementary material available at 10.1186/s13027-020-00333-4.

## Introduction

Traditionally cervical swabs have been placed in liquid media for transport. Due to the logistical difficulties such as spillage, flammability, and weight, adding to the risks and costs of liquid transport media, solid carriers consisting of chemically treated or untreated filter paper have been investigated for hrHPV testing. These filter paper cards are easy and safe to store and transport [1–13]. Recently we (Maurer, et.al.) designed and tested a new solid media transport card (POI card) that was compared to the established iFTA card from GE Healthcare. The new card performed equal to the iFTA card in terms of transfer of HPV DNA and sensitivity/specificity for CIN2+. In addition, the new card did not degrade in high humid environments like the iFTA card [14]. In a sub study (Luo et al.) provides data to suggest the iFTA may be a poorer transport vehicle than the new card when combined with the Cobas assay [15]. Therefore, in this trial we will evaluate the recently validated POI card for detecting HR-HPV in vaginal self-samples compared to self-collected samples transported in the standard PreservCyt liquid.

The Cobas 4800 HPV test, the first approved HPV assay for primary screening by US FDA, is a qualitative multiplex assay, providing specific genotyping information for HPV types 16 and 18, and then 12 other high-risk HPV types as a pooled result [16]. The SeqHPV test (BGI Shenzhen, Shenzhen, China) is a high throughput HPV genotyping assay based on multiplex PCR and next generation sequencing It is configured to detect 14 high risk types of HPV (16,18, 31, 33, 35, 39, 45, 51, 52, 56, 58, 59, 66 and 68). These characteristics make it well suited for centralized laboratory processing in high volume screening programs. The assay has been previously validated using SHENCCAST II specimens (Shenzhen Cervical Cancer Screening Trial [17]. Our study was designed to evaluate the performance of the Cobas 4800 HPV assay and SeqHPV for testing vaginal self-samples via either liquid or solid specimen transport method. Based on the multi-center population-based cervical cancer screening, we identified the concordance and the effectiveness in detecting high-risk HPV in different self-sample collection media.

## Materials and methods

### Study population and design

Between Aug 2016 to Aug 2018, a multi-center, population-based cross-sectional cervical cancer screening study [Chinese Multi-Center Screening Trial (CHIMUST)] was conducted in China.10,885 subjects were recruited from 15 screening sites in 7 provinces. Women in those sites were eligible if they were 25–59 years of age, sex-experienced but not pregnant, had no cervical cancer screening for at least 3 years, no prior hysterectomy, and no prior pelvic radiation. The study protocol was approved by the human subject review boards of the Peking University Shenzhen Hospital (PUSH), Shenzhen, China, and the Cleveland Clinic, Cleveland, USA. In addition, the study was registered with the Chinese Clinic Trial Registry (chiCTR-EOC-16008456), an international clinical trials registry platform approved by the WHO. Women with incomplete data were excluded from the analyses. This included: (1) women testing positive by any HPV test without having colposcopy and biopsies; (2) Specimen is not sufficient or no sample for an HPV test; (3) Failure of any HPV test.

### Study samples collection

Every woman had contributed two specimens, one was collected by herself (Self-Sample), and one was collected from the endocervix by a clinician (Direct-Sample). The self-collected sample was first applied to the solid media transport card (labeled as SC-sample), then the brush placed in 6 ml ThinPrep® PreservCyt® Solution (Hologic Bedford, MA, USA) (labeled as SL-sample). The brush was then agitated in 6 ml PreservCyt Liquid (as a split sample). Self-sampling instructions were provided by poster diagrams and personal instruction. The physician-collected samples were placed in 20 ml ThinPrep® PreservCyt® Solution (labeled as DL-sample). All samples were stored at room temperature and were tested with Cobas 4800 HPV test and SeqHPV test for HR-HPV within 2 months of collection. Patients testing HPV positive (self or direct), were recalled for colposcopy with directed and random 4 quadrants micro-biopsies plus endocervical curettage (ECC). Histology slides were interpreted by a gynecologic pathologist from PUSH (Author C.W). Immunochemical staining with p16 was selectively obtained to adjudicate difficult cases.

### The solid transport card management protocol

The new solid transport card consists of PK 226® paper (PerkinElmer, Greenville, SC) treated with a combination of a lysing solution and a dye (Hyde Biomedical Corporation, Wuhu, Anhui, China). The lysing solution contains an ionic detergent to lyse cell membranes and stabilize DNA as well. Similar, to the FTA card, the indicating dye changes color when the sample is applied. The sample area on the card was punched using a 5-mm Harris micro-punch (BSD, USA). Each card was manually punched 3 times and placed in a single well in a 96-well plate. Then they were all washed once using 100 μl of sterile water. The water is carefully removed with a sterile fine-tip pipette. The DNA elution is performed with 50 μl of sterile water at 56 °C for 30 min immediately followed by 95 °C for 15 min. in a heating block. The 96-well plate containing DNA elution and pieces of card are then centrifuged at 4000 rpm for 3 min and the eluted DNA is transferred into a new 96-well plate. When performing the assays, 5ul of DNA will be used in each well of the 96 well plates for PCR, for Cobas assay, and SeqHPV. Our prior study shows that 5ul is the standard volume used for the Cobas assay, and SeqHPV, and it has been thoroughly tested and demonstrated to be optimal with > 99% adequate specimens. (Any storage for future use will be at − 80 °C) [14, 15].

### High risk HPV detection

All samples were tested with Cobas 4800 HPV test and SeqHPV test for HR-HPV according to the manufacturer’s instructions. All the DL-Samples and SL-samples accepted by the PUSH lab were split, after thoroughly mixed by shaking, 1 cc used for Cobas 4800 and 1 cc for SeqHPV testing. After splitting, the physician-collected samples were processed for cytology interpretation. The Cobas 4800 system platform (Roche Molecular Diagnostics, Pleasanton, CA.), consists of the Cobas × 480 instrument and the Cobas z480 analyzer. We used two different specimen preparation procedures for the Cobas z480 that had previously been optimized. Nucleic acids used for the Cobas assay from the two liquid specimens (DL and SL), were prepared using Cobas × 480. The instrument could yield 50 μL of nucleic acid, eluted from 500 μL of ThinPrep solution used per subject. The eluted DNA solutions from the solid cards were prepared according to the Cobas4800 device “special instructions”. The “PCRONLY” program was performed using Cobas z480.5 μL DNA solution from the solid card samples were needed to ensure a sufficient sample (adequate DNA) was present for valid detection.

The “split sample” methodology we used, was used extensively in the 1990’s for the development of liquid based cytology [18]. The Both of HPV assays we studied were designed using a human β-globin gene (HBB, House keeping gene) as an internal control to identify false negatives caused by inadequate DNA or failed PCR.

### Cytology

Cytology using the Hologic I2 imager systems (computer assisted diagnosis) will be used for future research not for patient care in the current study.

### Statistical analysis

Data were entered in an ACCESS database specially designed for CHIMUST. To evaluate the effectiveness of different sample collection media for vaginal self-samples in population-based screening, we calculated sensitivities for detecting CIN2+ and CIN3+, the concordance in detecting high-risk HPV, and the differences in HPV assay performance using solid (filter paper cards) and liquid based specimen transport for the self-collected and physician collected cervico-vaginal specimens. Sensitivity and specificity of the Cobas and SeqHPV testing results in detecting high-risk HPV and CIN2 + were calculated using CIN2+ as the endpoint. McNemar’s Chi-square was performed to calculate differences between paired proportions at a probability level of 0.05. Agreement between self and direct samples was measured by absolute agreement and Kappa statistics (Cohen’s Kappa). Agreement between the solid transport media and liquid transport media for detecting HR-HPV in vaginal self-samples was also measured. All data were analyzed using SPSS 17.0.

## Results

### Demographic characteristics of participants

Ten thousand three hundred ninety-nine women had complete data. Mean age was 43.9 years. The return rate for colposcopy was 81.0%. 1.4% (141 patients) had CIN2+ and 0.5% (or 54 patients) had CIN3 + .101 (0.93%) women were dropped from the analysis due to HPV test failure. 6 (0.05%), 2 (0.02%), 29 (0.27%),34 (0.31%), 19 (0.17), and 19 (0.17) were missing Cobas-DL, Cobas-SL, Cobas-SC, Seq HPV-DL, SeqHPV-SL and SeqHPV-SC, respectively. Testing failure in both the assays were reported by the lab as inadequate DNA (Table supplement 1).

### HPV positivity in different collection media

Overall the HPV infection rates were 10.8% for Cobas and 10.9% for SeqHPV in clinician-collected samples (labeled as DL-sample), and 13.7% for Cobas and 11.6% for SeqHPV in self-samples placed in liquid medium (labeled as SL-sample), and 12.4% for Cobas and 11.6% for SeqHPV in self-samples collected on the solid transport card (labeled as SC-sample) (Table 1).

**Table 1:** The prevalence of HPV

HPV Screening tests	The prevalence of HPV	HPV Screening tests	The prevalence of HPV
Cobas-DL(doctor liquid)	10.8%	Seq--DL(doctor liquid)	10.9%
Cobas-SL (self liquid)	13.7%	Seq--SL(self liquid)	11.6%
Cobas-SC(self card)	12.4%	Seq-SC(self card)	11.6%

Note: *DL* Doctor liquid, endocervical samples in liquid media; *SL* Self liquid, cervicovaginal specimen in liquid media; *SC* Self card, cervicovaginal specimen in solid transport card

### HPV concordance for the two different self-sample collection media

Tables 2 and 3 show the agreement in HPV detection between the two different self-sample collection media (Solid media vs liquid media) was very good (Cobas HPV kappa = 0.86; SeqHPV kappa = 0.98).

**Table 2:** The concordance in detecting high-risk HPV between Cobas SL and SC

		Cobas-SL		% Positive Agreement	% Negative Agreement	% Overall Agreement	Kappa Value [95% CI]
		+	-				
Cobas-SC	+	1197	236	92.21	97.40	96.84	0.86 [0.85-0.88]
	-	92	8874

**Table 3:** The concordance in detecting high-risk HPV between Seq-SL and Seq-SC

		Seq-SL		% Positive Agreement	% Negative Agreement	% Overall Agreement	Kappa Value [95% CI]
		+	-				
Seq-SC	+	1185	24	97.85	99.73	99.52	0.98 [0.97-0.98]
	-	26	9164				

### Accuracy of detecting CIN2+, CIN3+ in different self-sample collection media

Tables 4 and 5 show the sensitivity and specificity of different self-sample collection media for CIN2+ and CIN3+ along with confidence intervals and *p*-values. Tested with Cobas, the sensitivity of Cobas-SC and Cobas-SL for CIN 2 + was 94.37 and 95.07%; and for CIN3+ was 96.30 and 96.30% respectively. The specificity of Cobas-SC, and Cobas-SL for CIN2+ was 87.74 and 88.35%; and for CIN3 was 88.04 and 86.65% respectively. Tested with SeqHPV, the sensitivity for CIN2+ of Seq-SC and Seq-SL was 95.77and 96.48%; and for CIN3+, both the SC and SL specimens had a sensitivity of 100%. The specificity for CIN2+ of Seq-SL and Seq-SC was 89.54 and 89.53%; and for CIN3+ was 88.84 and 88.82% respectively. For the two HPV assays, there was no significant difference in sensitivity for both the detection of CIN2+ and CIN3+ between SC and SL (*p* = 1.00). The card (SC) was significantly more specific than the self-liquid sample (SL) on the Cobas assay but similar with SeqHPV for both CIN2+ and CIN3+ (Tables 4 and 5).

**Table 4:** The sensitivity and specificity for **≥CIN 2** for the Cobas and SeqHPV assays

HPV tests	Sensitivity (95%CI)	*P* value	Specificity (95% CI)	*P* value	NPV ((95% CI)
Cobas--SL	95.07 (89.72, 97.82)	-	87.35 (86.68, 87.98)	-	99.92 (99.83,99.97)
Cobas—SC	94.37 (88.83, 97.36)	1.0	88.74 (88.11, 89.34)	<0.001	99.91 (99.82,99.96)
Seq--SL	96.48 (91.55, 98.70)	-	89.53 (88.92, 90.11)	-	99.95 (99.87,99.98)
Seq—SC	95.77 (90.63, 98.27)	1.0	89.54 (88.93, 90.12)	0.89	99.93 (99.85,99.97)

*Note: ≥ CIN2* Cervical intraepithelial neoplasia 2 or higher, *95%CI* 95% confidence interval

The comparison of solid vs. liquid transport media for detecting HR-HPV in vaginal self-collected specimens were calculated using the McNemar’s Chi-square test

**Table 5:** The sensitivity and specificity for **≥CIN 3** for the Cobas and SeqHPV assays

HPV tests	Sensitivity (95%CI)	*P* value	Specificity (95% CI)	*P* value	NPV (95% CI)
Cobas--SL	96.30 (86.16, 99.36)	-	86.65 (85.98, 87.30)	-	99.98 (99.91,100)
Cobas –SC	96.30 (86.16, 99.36)	1.0	88.04 (87.40, 88.66)	<0.001	99.98 (99.91,100)
SEQ --SL	100 (91.73, 100)	-	88.82 (88.10, 89.41)	-	100 (99.95,100)
SEQ –SC	100 (91.72, 100)	1.0	88.84 (88.21, 89.43)	0.78	100 (99.95,100)

*Note: ≥ CIN3* Cervical intraepithelial neoplasia 3 or higher

## Discussion

As noted in the introduction, the current study expands our prior work using “true” self-collections and only the POI card. We still used the split sample method to generate the SC (card) and the SL (liquid) samples. Our data shows that the POI Card performed well in this self-collection trial demonstrating equal sensitivity to liquid samples run both on Cobas and SeqHPV. We know from our prior work in SPOCCS III, that self-collected specimens will identify more HPV than direct endocervical specimens. This excess HPV found in the vagina is unassociated with CIN in the cervix [19]. Of interest it appears that more cellular DNA was washed from the brush in the secondary liquid split self-samples. This appears to have not affected the SeqHPV assay due to its analytical sensitivity but did the Cobas assay. In addition, even with a non-significant increase in sensitivity, since the SeqHPV assay has variable cut-points by HPV type based on the likelihood of causing high grade CIN, it did not affect specificity.

In 2016 we published our work developing a solid media transport card [14]. The new card project was initiated for several reasons: First we anticipated the need to collect thousands of samples per day and realized that the common alcohol (methanol, ethanol) containing liquid transport medias were a risk for children and spillage when home collections were involved. Second, the daily transport of these alcohol containing liquid specimens was complex and expensive. Third, a solid media transport seemed to be a good alternative but the commercial cards available at the time (iFTA, G.E. Healthcare) were expensive, especially for our medically underserved populations. In addition, in our initial trials the iFTA deteriorated in high humid environments.

After developing the POI card, we published a second study comparing liquid (SurePath) vs solid media (iFTA and POI cards) with Cobas. For this pilot study to control the self-collection we used a pseudo-self-collection technique (physician collects cervico-vaginal sample without using a vaginal speculum) [15]. We were both surprised and puzzled to discover the Cobas4800 test in combination with iFTA card (FTA) was inferior to the other samples studied (liquid self, POI card, direct liquid), both for the detection of HrHPV as well as CIN2/3+. This was especially surprising for the comparison “liquid self” to “FTA”, since the liquid self-sample was a secondary sample (split-sample) from FTA (the primary sample).

In a study from Sweden evaluating the HRVIR assay (laboratory developed test) compared to Cobas using FTA cards, the clinical sensitivity of the FTA card and HPVIR test was equivalent to Cobas, and the Cobas assay detected 63 of 67 women with CIN2+ [94.0% (95% CI = 85.2–98.1)] [20]. Likewise, Dong et.al. evaluated FTA and liquid samples with Cobas in a small study and also found good concordance. Interestingly, they found the same concordance for dry brush samples which for cost and simplicity is an important future direction [21].

In our trial when split samples were used, we have twice encountered discordant results, once with the primary card sample and once with the secondary liquid sample. Not with standing, we believe the strength of our work and the current literature supports the equivalency of solid media specimen transport, and liquid transport methodologies. The potential impact for non-liquid specimen transport on mass population-based screening is profound. Especially in current times of COVID, where self-collection methodologies will be preferable than bringing large populations together for move conventional cervical cancer screening events.

## Conclusions

We believe that the current study adds considerable power to the literature on specimen media comparisons, with 141 cases of CIN2+. The use of non-liquid forms of specimen transport, especially integrated with self-collection sampling screening programs appears to demonstrate equivalency and is an important area for future study and implementation.

## Supplementary Information

**Additional file 1: Table supplement 1.** Rate of HPV testing failure.

## Data Availability

All data generated or analyzed during this study are included in this article. The datasets used and/or analyzed during the current study are available from the corresponding author on reasonable request.
