# Supplementary material for: An evaluation of solid versus liquid transport media for high-risk HPV detection and cervical cancer screening on self-collected specimens
Source: Infect Agent Cancer. 2020 Nov 30;15:72. doi: 10.1186/s13027-020-00333-4 (PMC7706049; doi:10.1186/s13027-020-00333-4)
Supplement: Supplementary file 1 — Additional file 1: Table supplement 1. Rate of HPV testing failure. [file 13027_2020_333_MOESM1_ESM.docx]

**Table supplement 1**- Rate of HPV testing failure

| HPV screening test | No. (%) HPV testing falure |
| --- | --- |
| Cobas-DL | 0.05（6） |
| Cobas-SL | 0.02（2） |
| Cobas-SC | 0.27（29） |
| Seq-DL | 0.31（34） |
| Seq-SL | 0.17（19） |
| Seq-SC | 0.17（19） |
